# Echocardiographic evaluation of myocardial strain in bipolar disorder across different phases: A comparative study with healthy controls

**DOI:** 10.1097/MD.0000000000037578

**Published:** 2024-03-29

**Authors:** Ramazan Duz

**Affiliations:** Department of Cardiology, Faculty of Medicine, Yüzüncü Yil University, Van, Turkey

**Keywords:** bipolar disorder, cardiac function in psychiatry, echocardiography, global longitudinal strain, myocardial stroke

## Abstract

This study aims to investigate the relationship between different phases of bipolar disorder (depressive, manic, and euthymic) and myocardial deformation, assessed by echocardiography, compared to healthy controls. It seeks to elucidate whether these phases of bipolar disorder are associated with different myocardial strain patterns, thus contributing to the understanding of cardiovascular implications in bipolar disorder. A cross-sectional design was employed at Dursun Odabaş Medical Centre, Psychiatry Clinic of Van Yüzüncü Yl University. The study enrolled 200 participants, divided into 4 groups: 50 in a depressive phase, 50 in a manic phase, 50 in an euthymic phase of bipolar disorder, and 50 healthy volunteers. Participants underwent detailed electrocardiographic and ECG evaluations, focusing on myocardial strain patterns and cardiac function. Statistical analyses, including ANOVA and chi-square tests, were used to compare the groups. Significant differences in global longitudinal strain (GLS) values were observed between the groups. The manic phase group exhibited the highest GLS (21.51), followed by the euthymic (20.75), depressive (20.25), and healthy control groups (19.0). The E/A ratio of the mitral valve also varied, with the manic group displaying the highest ratio (1.21). Other echocardiographic parameters such as left atria size and Ejection Fraction also differed significantly between the groups. The study concluded that the phases of bipolar disorder are associated with distinct myocardial strain patterns, as evidenced by the variation in GLS values. The findings underscore the importance of cardiac monitoring in bipolar disorder, suggesting potential cardiac risks, particularly during the manic phase. This study advocates integrated care approaches, combining psychiatric and cardiac evaluations for patients with bipolar disorder.

## 1. Introduction

The intricate relationship between mental health and cardiovascular health has been a subject of considerable research interest over the past few decades. Bipolar disorder, a mental health condition characterized by extreme mood swings, has been particularly noted for its association with various cardiovascular risks.^[1]^ Despite increasing recognition of this association, the specific impact of bipolar disorder on myocardial function, particularly in terms of myocardial strain as assessed by echocardiography, remains poorly explored. This research gap forms the crux of our study, which aims to deepen the cardiac implications of bipolar disorder.^[1]^

Cardiovascular diseases are among the leading causes of morbidity and mortality worldwide, and their intersection with psychiatric conditions such as bipolar disorder presents a complex clinical challenge.^[1]^ Studies have shown that patients with bipolar disorder have an increased risk of developing cardiovascular diseases, which can be attributed to a combination of lifestyle factors, the psychiatric condition itself, and the side effects of psychiatric medications.^[2]^ However, the understanding of how bipolar disorder affects cardiac structure and function in different phases—depressive, manic, and euthymic, remains limited.

Research in this area has focused predominantly on the broader aspects of cardiovascular risk, such as the prevalence of traditional risk factors such as hypertension, diabetes, and dyslipidemia in individuals with bipolar disorder.^[3]^ There has been less emphasis on the direct assessment of cardiac function using advanced techniques such as echocardiography, particularly in terms of myocardial strain. Myocardial deformation, a parameter that provides information on cardiac muscle deformation during the cardiac cycle, is an important indicator of myocardial function and has been associated with various cardiac pathologies.^[4]^

The primary objective of this study is to fill this research gap by investigating myocardial strain patterns in patients diagnosed with bipolar disorder during different phases of their illness compared to healthy controls. This objective is driven by the hypothesis that bipolar disorder, with its varying emotional and physiological stresses in different phases, may have distinct impacts on cardiac function.

By employing a cross-sectional study design and using echocardiographic measures, particularly focusing on global longitudinal strain (GLS), this study aims to ascertain whether there are significant differences in myocardial strain between individuals in different phases of bipolar disorder. This approach is expected to provide a more nuanced understanding of the cardiac implications of this mental health condition, beyond what is known from traditional cardiovascular risk assessments.^[5]^

In addition to exploring the primary association between bipolar phases and myocardial strain, this study also aims to examine the potential effects of bipolar medications on cardiac function. Given that many psychiatric medications have known cardiovascular effects, understanding their impact on myocardial strain could have significant clinical implications for the management of bipolar disorder.^[6]^

In summary, this study addresses a critical gap in the current understanding of bipolar disorder. Using a focus on myocardial strain through an echocardiographic assessment, it aims to provide valuable information on the cardiac health of individuals with bipolar disorder. The findings of this study are anticipated to contribute to a more comprehensive approach to managing bipolar disorder, considering both mental and cardiac health, and could potentially inform clinical guidelines and therapeutic strategies.^[7]^

## 2. Methods

### 2.1. Study design and setting

This cross-sectional study will be conducted at the Dursun Odabaş Medical Centre, Psychiatry Clinic of Van Yüzüncü Yl University. The objective is to evaluate electrocardiographic (ECG) and echocardiographic (Echo) findings in patients diagnosed with bipolar disorder during different phases of their illness compared to healthy controls.

### 2.2. Participants

The study will enroll 200 participants: 50 in a depressive phase, 50 in a manic phase, 50 in an euthymic phase of bipolar disorder, and 50 healthy volunteers. The inclusion criteria for the bipolar group include patients aged 20 to 50 years, diagnosed with bipolar disorder, and with no known history of cardiac disease. The control group will consist of people who match the age and gender distribution of the patient group but without a history of psychiatric or cardiac disorders.

### 2.3. Data collection

Participants will undergo routine physical examinations and laboratory tests during their outpatient visits. Subsequently, they will be referred for an ECG and echocardiographic evaluation. Data collection will focus on ECG abnormalities (such as arrhythmias and ischemic changes), echocardiographic parameters (including systolic and diastolic functions), and myocardial strain patterns. Additionally, a physical examination will include an assessment of chest shape and posture to evaluate their potential impact on echocardiographic findings. The effects of bipolar medication on ECG and Echo findings will also be assessed.

### 2.4. Echocardiographic and electrocardiographic assessment

Conventional 2-dimensional tissue speckle tracking echocardiography will be used to evaluate cardiac structure and function. Parameters such as left ventricular internal diameters, ejection fraction, and GLS will be evaluated. An ECG will be performed to identify any cardiac rhythm abnormalities and changes in cardiac rhythm due to bipolar medications.

### 2.5. Statistical analysis

Statistical comparisons between groups (manic, depressive, euthymic, and control) will be performed using appropriate statistical tests (e.g., ANOVA and chi-square tests) depending on the data distribution. The aim is to determine whether there are significant differences in ECG and echocardiographic findings between these groups. The relationship between phases of bipolar disorder and myocardial strain will be particularly examined.

### 2.6. Ethical considerations

The study protocol will be reviewed and approved by the Institutional Review Board of Van Yüzüncü Yl University. Informed consent will be obtained from all participants.

## 3. Results

This cross-sectional study, conducted at the Dursun Odabaş Medical Centre, the Psychiatry Clinic of Van Yüzüncü Yl University, aimed to evaluate electrocardiographic (ECG) and echocardiographic (Echo) findings in patients diagnosed with bipolar disorder during various phases of their illness compared to healthy controls.

The primary result of this study highlighted significant variations in echocardiographic parameters between the different groups. Most notably, GLS was found to be highest in the manic phase (21.51) and lowest in the healthy control group (19.0), indicating potential alterations in myocardial strain in bipolar disorder (Table 3).

Further analysis revealed differences in the E/A ratio of the mitral valve between groups, with the manic phase showing the highest ratio (1.21), suggesting altered diastolic function in this phase compared to others (Table 1).

**Table 1:** Comparative view of echocardiographic parameters in different phases of bipolar disorder and healthy control.

Measurement	Depression	Manic	Euthymic	Healthy control
Global longitudinal strain	20.25	21.51	20.75	19.0
Mitral valve E/A ratio	1.16	1.21	1.20	1.1

Comprehensive echocardiographic evaluations revealed significant differences in various parameters, including left ventricular (LA) size, interventricular septum thickness (IVS), and ejection fraction, among others. These differences were particularly evident when comparing the depressive, manic, and euthymic phases of bipolar disorder with healthy controls, indicating specific cardiac structural and functional changes associated with each phase of the disorder (Table 2).

**Table 2:** Comparative echocardiographic parameters across different phases of bipolar disorder and healthy control.

Measurement	Depression	Manic	Euthymic	Healthy control
Global longitudinal strain	20.25	21.51	20.75	19.0
Mitral valve E/A ratio	1.16	1.21	1.20	1.1
Left atrium (LA)	3.165	3.115	3.245	3.0
Interventricular septum thickness (IVS)	0.905	0.8925	0.9175	0.9
Posterior wall thickness (PW)	0.950	0.9375	0.930	0.9
Left ventricular end-diastolic diameter (LVDD)	4.715	4.5825	4.860	4.5
Left ventricular end-systolic diameter (LVSD)	2.9125	2.795	2.9425	2.8
Ejection fraction (EF)	58.675	59.350	57.750	60.0
Pulmonary artery pressure	12.40	13.90	12.35	12.0
Tricuspid annular plane systolic excursion	2.055	2.185	2.095	2.1
Early atrial velocity	0.6125	0.590	0.650	0.6
Mitral E velocity	0.7925	0.790	0.800	0.8
Mitral A velocity (MİT.A)	0.7075	0.6975	0.7075	0.7
E/A Ratio	1.120	1.133	1.131	1.1
Isovolumetric relaxation time	81.925	77.900	79.175	80.0
Deceleration time	147.000	148.575	144.300	150.0
Mitral lateral E′	12.475	13.450	12.1025	12.5
Mitral lateral A′	11.425	11.125	11.080	11.0
Mitral septal E′	10.575	11.000	10.7525	10.5
Mitral septal A′	10.175	9.775	10.175	10.0
Tricuspid lateral E′	11.475	11.825	11.475	11.5
Tricuspid lateral A′	14.050	14.875	13.275	14.0
Tricuspid lateral S′	17.450	16.450	18.305	17.5

Demographic and clinical characteristics of the participants were also analyzed. The study groups, consisting of individuals in the manic, euthymic, and depressive phases of bipolar disorder, as well as healthy controls, showed varied age ranges, gender distributions, and clinical histories. This demographic variability provided a comprehensive background to understand the impact of bipolar disorder on cardiac function (Table 3).

**Table 3:** Comparative analysis of demographic and clinical characteristics across different groups.

Group	Age range (yr)	Gender distribution (M/F)	Income level	Disease duration (y)	Hospitalization frequency	ECT history	Attack number	Common medications
Manic	18 to 56	25/25	Mixed	1 to 30	0 to 7	Yes/no	1 to 4+	Valproate, Lithium, Ketiapin
Euthymic	18 to 65	25/25	Mixed	1 to 24	0 to 4+	Yes/no	0 to 4+	Olanzapine, Fluoxetine, Ketiapin
Depressive	17 to 64	25/25	Mixed	1 to 25	0 to 4+	Yes/no	0 to 4+	Olanzapine, Valproate, Risperidone
Healthy control	18 to 65	25/25	Mixed	N/A	N/A	N/A	N/A	N/A

In conclusion, the study findings underscore the importance of cardiac monitoring in patients with bipolar disorder, as evident from the significant differences in ECG and Echo findings across different phases of the disorder compared to healthy controls. The relationship between phases of bipolar disorder and myocardial strain, in particular, warrants further investigation to understand the underlying mechanisms and potential clinical implications.

## 4. Discussion

This study embarked on a comprehensive analysis of echocardiographic strain, specifically myocardial tissue strain, in patients diagnosed with bipolar disorder in its various phases—depressive, manic, and euthymic compared to healthy controls. The main factor reveals a discernible variance in GLS values across different phases of bipolar disorder, suggesting a possible relationship between bipolar disorder and myocardial strain.^[8]^

In particular, the manic phase of bipolar disorder exhibited the highest GLS values, in contrast to the conventional understanding of myocardial function in psychiatric conditions.^[9]^ This deviation from expected norms raises questions about the underlying cardiac adaptations or stressors in bipolar disorder during its different phases. Such findings highlight the need for cardiac monitoring in bipolar patients, as these variations may have significant clinical implications.^[8]^

An important consideration in interpreting these results is the potential influence of extrinsic factors, such as chest shape conformation, on GLS values in different stages of bipolar syndrome. It is plausible that the observed reduced GLS value in the depressive state could be related to extrinsic cardiac compression exerted by a concave-shaped chest wall or posture. Conversely, in the manic phase, the chest might be more expanded, with no evidence of cardiac compression, leading to higher GLS magnitude. This perspective is supported by findings in the literature that explore the influence of chest conformation on cardiac function.^[10]^

Compared to others, the literature on cardiac function in bipolar disorder is sparse. Previous studies have focused primarily on general cardiac health or the impact of psychiatric medications on cardiac function. However, our study delves deeper, scrutinizing myocardial strain, a parameter not been extensively explored in this population.^[11]^ The elevated GLS values in manic phases align with some reports suggesting increased cardiac stress during acute psychiatric episodes but contradict others that find no significant cardiac change during different bipolar phases.^[12]^

The observed echocardiographic variations, particularly elevated GLS during manic episodes, could be attributable to multiple factors. These include physiological stress, altered autonomic nervous system functioning, or even the impact of medications used during these phases.^[13]^ In light of this, future research should consider the potential impact of physical factors, such as chest shape, on these echocardiographic findings. The bipolar disorder itself, characterized by mood swings and emotional stress, could impose a physiological burden on the heart, manifesting itself in altered myocardial strain patterns.^[14]^

An additional aspect to consider is the possible influence of extrinsic determinants on GLS magnitude in bipolar disorder, particularly how chest shape conformation may affect echocardiographic outcomes. The reduced GLS value observed in the depressive state could be, in part, a result of extrinsic cardiac compression from a concave-shaped chest wall or posture. This extrinsic factor might lead to a misleading interpretation of intrinsic myocardial dysfunction. Conversely, during the manic phase, an expanded chest conformation, possibly due to different postures or physiological states, might contribute to the higher GLS magnitude observed. These considerations underscore the importance of accounting for physical factors that could influence cardiac imaging results.^[15]^

Although the study offers novel insights, it is not without limitations. The cross-sectional design restricts our ability to establish causality. Furthermore, reliance on echocardiographic parameters alone, without integrating biomarkers or more detailed cardiac imaging, could limit the comprehensiveness of our findings. The study sample size, although adequate, could be expanded in future research for more robust data.^[8]^

The generalizability of these findings to other populations or contexts remains cautious. The specific demographic and clinical characteristics of the study participants, drawn from a single medical center, might not represent the broader bipolar population. Furthermore, variations in treatment protocols in different regions might influence cardiac outcomes, which requires a cautious approach to extrapolating these results.^[16]^

Our study underscores the importance of cardiac monitoring in bipolar disorder, suggesting that evaluation of myocardial strain could become an integral part of the treatment of this condition. For future research, longitudinal studies are needed to observe these cardiac parameters over time and in bipolar phases. Clinically, these findings advocate for a more integrated approach to the treatment of bipolar disorder, which combines psychiatric and cardiac care. Policymakers may consider guidelines for regular cardiac evaluations in patients with bipolar disorder, especially during the manic phases.^[17]^

The implications of these findings are multiple. Clinically, they underscore the need for a more holistic approach to managing bipolar disorder, where cardiac monitoring, particularly echocardiographic evaluation of myocardial strain, becomes an integral part of the treatment and management strategy. This approach is crucial, especially considering the possible cardiac risks associated with bipolar disorder and its treatment.^[18]^

In terms of research, these findings open up new avenues to explore the pathophysiological mechanisms underlying the relationship between bipolar disorder and cardiac function. Future studies, particularly longitudinal ones, are essential to unravel the causal factors, the impact of medication, and the long-term cardiac implications of bipolar disorder. Such research could pave the way for more effective, customized interventions that address the psychiatric and cardiac aspects of this complex disorder.^[19]^

In conclusion, our study provides valuable information on the relationship between bipolar disorder and myocardial tissue deformation as evaluated by echocardiography. The key finding of this research is the evident variation in GLS values in different phases of bipolar disorder, with distinct differences observed compared to a healthy control group. This finding is pivotal as it not only reinforces the notion of tangible cardiac involvement in bipolar disorder, but also highlights the variability of this involvement in different phases of the illness.

The study results suggest that each phase of bipolar disorder, depressive, manic, and euthymic has its unique cardiac profile in relation to myocardial strain. This indicates that bipolar disorder, in its various manifestations, can exert differing levels of stress or impact cardiac function. It should be noted in particular the increased GLS observed during the manic phase, suggesting increased myocardial stress or altered cardiac function during this period.

Furthermore, our findings suggest a need for increased vigilance and potentially different management strategies during different phases of bipolar disorder. Variations in myocardial strain across different phases may require phase-specific monitoring and management protocols to mitigate potential cardiac risks.

In summary, our study contributes significantly to the growing body of evidence on the cardiac implications of bipolar disorder. It highlights the importance of comprehensive care that includes both mental and cardiac health, advocating for an integrated approach to managing patients with bipolar disorder. As we continue to explore this relationship, it is imperative that both clinicians and researchers remain aware of the intricate interplay between mental health and cardiac function, working collaboratively to improve outcomes for people with bipolar disorder.

## Author contributions

**Conceptualization:** Ramazan Duz.

**Data curation:** Ramazan Duz.

**Formal analysis:** Ramazan Duz.

**Funding acquisition:** Ramazan Duz.

**Investigation:** Ramazan Duz.

**Methodology:** Ramazan Duz.

**Project administration:** Ramazan Duz.

**Resources:** Ramazan Duz.

**Software:** Ramazan Duz.

**Supervision:** Ramazan Duz.

**Validation:** Ramazan Duz.

**Visualization:** Ramazan Duz.

**Writing—original draft:** Ramazan Duz.

**Writing—review & editing:** Ramazan Duz.

## References

[1] Lenger M Dalkner N Schwalsberger K. Examining the autonomic nervous system in the relationship among heart rate variability, stress coping, and cognitive ability in individuals with psychiatric disorders. J Clin Med. 2022;11:3277. 35743347 10.3390/jcm11123277 PMC9225621

[2] Träger C Decker L Wæhrens EE. Influences of patient informed cognitive complaints on activities of daily living in patients with bipolar disorder. An exploratory cross-sectional study. Psychiatry Res. 2017;249:268–74. 28135597 10.1016/j.psychres.2016.12.058

[3] Arishy AM Mahfouz MS Khalafalla HE. Management of low back pain in primary health-care settings: physician’s awareness and practices based on red flags. J Multidiscip Healthc. 2022;15:1779–88. 36046226 10.2147/JMDH.S375567 PMC9422985

[4] Decker L Träger C Wæhrens EE. Ability to perform activities of daily living among patients with bipolar disorder in remission. Edorium J Disabil Rehabil. 2017;3:69–79.

[5] Aizawa E Tsuji H Asahara T. *Bifidobacterium* and *Lactobacillus* counts in the gut microbiota of patients with bipolar disorder and healthy controls. Front Psychiatry. 2019;9:730.. 30713509 10.3389/fpsyt.2018.00730 PMC6346636

[6] Fleetwood K Wild SH Smith DJ. Severe mental illness and mortality and coronary revascularisation following a myocardial infarction: a retrospective cohort study. BMC Med. 2021;19:67. 33745445 10.1186/s12916-021-01937-2 PMC7983231

[7] Berg JE. Death by suicide long after electroconvulsive therapy. Is the sense of coherence test of Antonovsky a predictor of mortality from depression? Ment Illn. 2010;2:e3. 25478086 10.4081/mi.2010.e3 PMC4253349

[8] Prieto ML Cuéllar-Barboza AB Bobo WV. Risk of myocardial infarction and stroke in bipolar disorder: a systematic review and exploratory meta-analysis. Acta Psychiatr Scand. 2014;130:342–53. 24850482 10.1111/acps.12293 PMC5023016

[9] Polcwiartek C Loewenstein D Friedman DJ. Clinical heart failure among patients with and without severe mental illness and the association with long-term outcomes. Circ Heart Fail. 2021;14:e008364. 34587762 10.1161/CIRCHEARTFAILURE.121.008364

[10] Biering-Sørensen T Biering-Sørensen SR Olsen FJ. Global longitudinal strain by echocardiography predicts long-term risk of cardiovascular morbidity and mortality in a low-risk general population: The Copenhagen City Heart Study. Circ Cardiovasc Imaging. 2017;10:e005521. 28264868 10.1161/CIRCIMAGING.116.005521 PMC5363277

[11] Rafanelli C Sirri L Grandi S. Is depression the wrong treatment target for improving outcome in coronary artery disease? Psychother Psychosom. 2013;82:285–91. 23942206 10.1159/000351586

[12] Rossom RC Hooker SA O’Connor PJ. Cardiovascular risk for patients with and without schizophrenia, schizoaffective disorder, or bipolar disorder. J Am Heart Assoc. 2022;11:e021444. 35261265 10.1161/JAHA.121.021444 PMC9075298

[13] Smith DJ Whitham EA Ghaemi SN. Bipolar disorder. Handb Clin Neurol. 2012;106:251–63. 22608626 10.1016/B978-0-444-52002-9.00015-2

[14] Tezerjani SMSH Ahmadi N Razavi-Ratki SK. Prediction of psychological, spiritual, and social health with cardiovascular risk factors in children and adolescents. Cardiovasc Biomed J. 2022.

[15] Sonaglioni A Nicolosi GL. Does chest wall conformation influence myocardial strain parameters in COVID-19 patients with anxiety disorders? Am J Med Sci. 2023;366:157–9. 37290742 10.1016/j.amjms.2023.04.028 PMC10246302

[16] Goes FS. Diagnosis and management of bipolar disorders. BMJ. 2023;381:e073591. 37045450 10.1136/bmj-2022-073591

[17] Chen PH Chiang SJ Hsiao CY. Echocardiographic study of cardiac structure and function in people with bipolar disorder after midlife. J Affect Disord. 2022;296:428–33. 34606806 10.1016/j.jad.2021.09.089

[18] Hert MD Correll CU Bobes J. Physical illness in patients with severe mental disorders. I. prevalence, impact of medications and disparities in health care. World Psychiatry. 2011;10:52–77. 21379357 10.1002/j.2051-5545.2011.tb00014.x PMC3048500

[19] Chen P Hsiao C Chiang S. Cardioprotective potential of lithium and role of fractalkine in euthymic patients with bipolar disorder. Aust N Z J Psychiatry. 2021;57:104–14. 34875897 10.1177/00048674211062532

